# Outcome of highly active antiretroviral therapy in HIV- infected Indian children

**DOI:** 10.1186/s12879-014-0701-2

**Published:** 2014-12-24

**Authors:** Aparna Mukherjee, Nipam Shah, Ravinder Singh, Madhu Vajpayee, Sushil K Kabra, Rakesh Lodha

**Affiliations:** Departments of Pediatrics and Microbiology, All India Institute of Medical Sciences, Ansari Nagar, New Delhi, 110029 India

**Keywords:** HIV, Children, Highly active antiretroviral therapy, Long-term outcome

## Abstract

**Background:**

With the advent of highly active antiretroviral therapy (HAART), HIV infection has become a chronic condition in children with improved survival and quality of life. Reports on long term effectiveness of non-nucleoside reverse transcriptase inhibitor based HAART in HIV-infected children in developing countries are limited.

**Methods:**

A chart review was conducted and children who received at least six months of HAART between 2004–2011 at All India Institute of Medical Sciences (AIIMS), Delhi were included. The clinical, immunological and virological responses to HAART were documented. Factors predicting non-adherence and non-response to treatment were described.

**Results:**

One seventy five children (boys: 74.9%) were included in the study, with a median follow up of 43 (IQR:17, 68) months. The median age at diagnosis was 119 (IQR: 75, 156) months. The median CD4 count at start of HAART was 340 cells/μL (IQR: 185,704), which increased to 924 cells/μL (IQR:591,1278) at 48 months after HAART and plateaued at 749 (IQR: 542,1056) cells/ μL after 90 months of therapy. The weight for age (WAZ) and height for age (HAZ) z score both showed improvement with time after HAART initiation [baseline: WAZ −2.8 (IQR:-4,-1.6), HAZ −2.1 (IQR:-3.4,-0.69); at 42 months of therapy: WAZ −1.2 (IQR:-2.1, 0.01), HAZ −0.75(IQR:-1.6,-0.37)]. Adverse events were reported in 21 (12%) children. Non-adherence to therapy, treatment failure and death were noted in 35 (20%), 9 (5.1%) and 6 (3.4%) children respectively.

**Conclusions:**

Our experience shows that HAART in HIV-infected children is effective, safe and is associated with good immunological and virological response as well as improvement in growth parameters.

**Electronic supplementary material:**

The online version of this article (doi:10.1186/s12879-014-0701-2) contains supplementary material, which is available to authorized users.

## Background

Highly active anti-retroviral therapy (HAART) has been reported to be efficacious in terms of hastening immunological recovery and enhancing growth in HIV-infected children across the world. Survival has greatly improved with the advent of HAART in children. There are reports of positive influence of HAART on the overall quality of life of children living with HIV/AIDS [1]-[7]. There is paucity of studies reporting long term outcomes in children receiving non-nucleoside reverse transcriptase inhibitor (NNRTI) based HAART. Recently Phongsamart et al. have reported the long term outcomes in Thai HIV-infected children receiving ART; the median follow up was 2.9 years [8]. The authors concluded that immune reconstitution occurred in nearly 80% children. In a retrospective analysis of data, after 24 months of HAART, one in three children was likely to develop treatment failure [9]. In the ARROW trial, excellent outcomes were reported in children receiving NNRTI based or 3NRTI based regimen [10].

There is paucity of reports of long-term outcomes in HIV- infected children receiving NNRTI based regimen from Indian sub-continent. We report here the experience with use of HAART in HIV infected children who have been on long term follow up in an Indian center.

## Methods

We conducted a review of case records of HIV- infected children who received HAART in the Department of Pediatrics, All India Institute of Medical Sciences, Delhi, between 2004 to 2011. The study protocol of this retrospective review was approved by the Institute Ethics Committee (Ref No IEC/NP-354/2013). As this was a retrospective review of information in the case records, informed consent was not taken.

### Inclusion criteria

HIV-infected children, 1–15 years of age, who had received HAART for at least 6 months were included.

### Diagnosis

HIV infection was confirmed using three sets of Enzyme immunoassays (EIA) test for HIV-1 according to National AIDS Control Organization (NACO) guidelines [11]-[13]. In children less than 18 months age, diagnosis of HIV infection was made on the basis of two positive HIV-DNA PCR tests [13].

The routine diagnostic work-up for all patients included blood tests for serum biochemistry (renal and liver function tests), complete hemogram, CD4 cell count for all and also CD4 percentage for children ≤ 5 years. The CD4 was measured by flow cytometry (BD FACS Caliber, San Diego, USA). HIV RNA viral load was available for some subjects at different points of follow-up. Viral load estimation was done by real time PCR (COBAS TaqMan, Roche Diagnostics, Indiana, USA); the lower limit for detection was 47 copies/mL.

Based on clinical profile, a targeted search for an opportunistic infection was performed. Clinical and immunological stage was assigned to HIV children based upon the WHO clinical and immunological criteria for HIV staging [13]. Indications to start antiretroviral therapy were based on available guidelines [13]-[15].

### Treatment regime

The preferred first line antiretroviral drugs were combination of 2 nucleoside reverse transcriptase inhibitors (NRTI) + 1 non-nucleoside reverse transcriptase inhibitor (NNRTI): zidovudine(AZT) + lamivudine (3TC) + nevirapine (NVP) or efavirenz (EFV) or stavudine(d4T) + 3TC + NVP or EFV. In patients with HIV-TB co-infection, efavirenz based regimen was preferred in children > 3 years in view of interaction between nevirapine and rifampicin; in children below three years of age nevirapine was continued with rifampicin. Co- trimoxazole prophylaxis was administered according to available guidelines [13].

### Follow-up and monitoring

All the patients were followed every 3 months. During each follow up visit, weight and height were recorded, adherence to treatment was checked, symptoms were noted, physical examination was done, and side effects were recorded. Complete blood count, serum chemistry and CD4 counts were monitored every 6 months. Adherence to treatment was monitored by interviewing the child/guardian at each visit and by pill count. Non-adherence was defined as missing of three or more doses in a period of 30 days.

### Data collection

Data from the patients’ clinical records (obtained from the pediatric OPD medical record room) were recorded in the structured sheets and entered in the Microsoft Access 2010 to form an electronic database. A retrospective data auditing was performed to ensure accuracy. The primary outcomes of interest in this study were response to HAART in terms of immunological and clinical response, all cause mortality rate and treatment failure. The treatment failure was defined as per NACO guidelines [13]. Clinical failure was defined as the appearance or reappearance of WHO clinical stage 3 or stage 4 events after at least 24 weeks on HAART in a treatment-adherent child. Immunological failure was defined as developing or returning to the following age-related immunological thresholds after at least 24 weeks on HAART, in a treatment-adherent child:

- CD4 count of <200 cells/mm^3^ or %CD4 <10 for a child =2 years to <5 years of age
- CD4 count of <100 cells/mm^3^ for a child 5 years of age or older.

Virological failure was defined as a persistent viral load above 5000 RNA copies/mL on two consecutive measurements, after at least 24 weeks on HAART, in a treatment-adherent child. Death was ascertained either from the hospital records or from the telephonic conversation with the relatives. The clinical response to HAART was determined by decrease in the number of episodes of illness. Immunological response was based on the CD4 values at different time points. The secondary outcomes assessed were change in anthropometric values in terms of z scores at different time points and changes in blood parameters i.e. hemoglobin, erythrocyte sedimentation rate (ESR) over time. We also attempted to determine the factors associated with non-adherence and treatment failure.

### Statistical methods

The electronic data were exported into the Stata software, version 9.0 (StataCorp, College Station, TX, USA) for statistical analysis. The z scores for weight for age and height for age were calculated using the statistical package EPI-INFO 2000 (Centers for Disease Control and Prevention, USA). Logistic regression model was used to determine the factors responsible for non-adherence and also non-response to treatment. A generalized estimating equation model was tested to look into the association between average change of CD4 count over 48 month of HAART and other factors like age at diagnosis, gender, and average change of anthropometric parameters.

## Results

We screened records of 400 children; 175 children met the study criteria; these children had received HAART for atleast 6 months. The median (IQR) duration of follow up was 43 (IQR: 17, 68) months. Since 2005, six (3.4%) children died after initiating HAART and 20 (11.4%) children were lost to follow up. The median interval between initiation of HAART and death/ lost to follow up was 24 (IQR: 12,24) months.

### Baseline characteristics

The baseline characteristics of patients on HAART are mentioned in Table 1. The median age at diagnosis was 119 months (IQR: 75,156 months) and there was a predominance of boys (74.9%).

**Table 1:** **Baseline characteristics (clinical and demographic)**

Characteristics	Values
Age (median, IQR)	119 (75–156) months
Boys	131 (74.9)
Lost one parent	71 (40.5)
Lost both parents	33 (18.9)
Age at diagnosis (median, IQR)	58 (29,96) months
Mode of transmission	
Perinatal	142 (81.2)
Blood transfusion	6 (3.4)
Uncertain	27 (15.4)
WHO clinical stage at diagnosis	
1	49 (28)
2	50 (28.6)
3	56 (32)
4	20 (11.4)
WHO immune stage at diagnosis, n = 158	
None	31 (19.6)
Moderate	12 (7.6)
Advanced	25 (15.8)
Severe	90 (57)
WAZ before starting HAART	
≥−2 z	136 (77.7)
< −2 to> −3	14 (8)
≤−3	29 (14.3)
HAZ before starting HAART	
≥−2 z	148 (84.6)
< −2 to> −3	9 (5.1)
≤−3	18 (10.3)
Received BCG	109 (62.3)
History of contact with TB	60 (34.3 )
TB disease diagnosed before initiating HAART	47 (26.9 )
TB developed after initiating HAART	15 (8.6)

All values are n (%) unless specified. IQR: interquartile range, WHO: World Health Organization, WAZ: weight for age z score, HAZ : height for age z score, BCG: Bacillus Calmette Guerin, HAART: highly active anti-retroviral therapy, TB: tuberculosis.

At diagnosis, the most common clinical presentations were recurrent fever (87 children, 49.7%) and failure to thrive (70 children, 40%). Only 15% of HIV-infected children, who were subsequently initiated on HAART, were asymptomatic at presentation. Tuberculosis (47 children) was the most common opportunistic infection (OI) in these patients before starting HAART. After starting HAART, 15 (8.6%) children developed tuberculosis. Diagnosis of TB was clinico-radiological. AFB/ Mycobacterium tuberculosis was positive in eight children before starting HAART and only one child after starting HAART. Other OIs observed before starting HAART were cryptosporidiosis (4 children), *Herpes simplex* (7 children), candidiasis (4 children), *Isospora belli* (1 children) and Cytomegalovirus (2 children) infection.

Table 2 shows the immunological, virological and treatment parameters at the initiation of HAART. The median CD4 count at start of HAART was 340 cells/μL (IQR: 185, 704).

**Table 2:** **Immunological and treatment parameters at the initiation of therapy**

Parameter	Values
Duration of follow up while on HAART, months, median (IQR)	43 (17,68)
CD4 count, cells/μL, median (IQR)	340 (185,704)
Age <2 years (n = 27)	424 (304,729)
Age 2-5years (n = 20)	460 (260,871)
Age >5 years (n = 128)	288 (163,639)
CD4%, mean (SD)	13.3 (10.7)
Baseline regime of HAART, n (%)	
d4T+3TC+NVP	141 (80.6)
d4T+3TC+EFV	19 (10.8)
AZT+3TC+NVP	11 (6.3)
AZT+3TC+EFV	4 (2.3)

IQR: interquartile range.

HIV: human immunodeficiency virus, HAART: highly active anti-retroviral therapy.

d4T: stavudine, 3TC: lamivudine, AZT: zidovudine, NVP: nevirapine, EFV: efavirenz.

Response to anti-retroviral therapy: There was improvement in clinical symptoms with a decline in the number of episodes of clinical events after starting HAART. The number of episodes of upper respiratory tract infection over 6 months reduced from 5.5 events per 100 child- months in the first six months after starting HAART to 2.9 events per 100 child-months after 48 months of therapy. Similarly incidence of lower respiratory tract infections declined from 3.3 events per 100 child-months in the first six months after initiation of HAART to 1.4 events per 100 child-months at 48 months of therapy, febrile episodes due to any cause decreased from 3.9 to 2.1 events per 100 child-months. There was no significant difference in incidence of events like thrush, herpes infection or seizures (data not shown).

**Figure 1:**
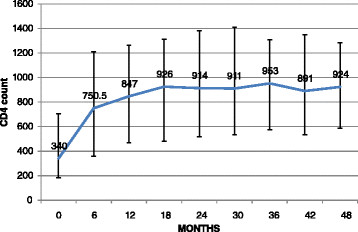
**Change in median CD4 count (cells/μL) with IQR over 48 months of HAART.**

There was good immunological and virological response to HAART. The median CD4 count increased from 340 cells/μL (IQR: 185,704) at initiation to 924 cells/μL (IQR: 591,1278) at 48 months after HAART (p < 0.001) and remained at 749 (IQR: 542, 1056) calls/ μL after 90 months of therapy. Figure 1 shows the trend in CD4 count from baseline to 48 months after HAART. The median CD4 count gradually peaked at 18 months post HAART and maintained a plateau thereafter upto 48 months. For children diagnosed before five years of age, the median CD4 percentage increased from 13% (IQR: 8.4, 21) at baseline to 29.3% (IQR: 19, 31) 48 months after initiation of HAART, p = 0.02. Viral load was available in 76 children. After a median duration of 34.4 months (IQR: 10.4, 47) of HAART the median viral load documented was 400 (IQR: 47, 958)RNA copies/mL; 49 (66%) children had undetectable viral load.

**Figure 2:**
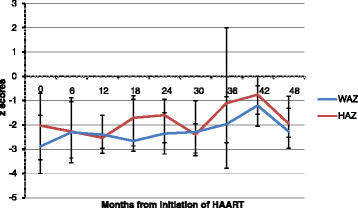
**Changes of weight for age (WAZ) and height for age (HAZ) z‐ scores over 48 months from the initiation of HAART.**

Anthropometric measures too showed improvement over time. The median WAZ changed from −2.8 (IQR: −4,-1.6) at the start of therapy to −1.2 (IQR: −2.1, 0.01) at 42 months post initiation of HAART, p = 0.04 (Figure 2). Median HAZ changed from −2.1 (IQR: −3.4,-0.69) at initiation to −0.75 (IQR:-1.6,-0.37) after 42 months of HAART, p = 0.04 (Figure 2). There were changes in the hematological parameters like hemoglobin and ESR in response to HAART. The mean (SD) hemoglobin increased from 9.3 (1.9) g/dL at baseline to 12.9 (1.3) g/dL at 84 months post HAART, p = 0.003; whereas ESR decreased from the mean (SD) of 41.9 (25) mm in 1^st^hr to 27.6 (17.1) mm in 1^st^ hr at 84 months of HAART, p = 0.1.

It was observed that the overall average change in CD4 count over 48 months of therapy did not have any significant association with the average change in weight for age and height for age z-scores. However, the average change in CD4 count was significantly associated with gender (p = 0.02), being more in female gender and also with age at diagnosis – the change being more for younger age at diagnosis (p = 0.02).

### Predictors of response to anti-retroviral therapy

Most of the children (94.9%) in this cohort responded well to HAART. Overall only 9 (5.1%) children were suspected of having treatment failure, of which clinical failure was seen in 4 children, immunological failure in 2 children and virological failure in 3 children. The median time to treatment failure from initiation of HAART was 43 (IQR: 18, 54) months. In a logistic regression model, treatment failure could not be predicted by factors like age at diagnosis, gender, initial CD4 count and duration of HAART (Table 3).

**Table 3:** **Predictors of treatment failure, by logistic regression model**

Characteristics	Odds ratio (95% CI)
Male gender	0.64 (0.07 – 6.16)
Age at diagnosis, months	0.99 (0.98 – 1.0)
CD4 count at initiation of HAART	0.99 (0.99 – 1.0)
Duration of HAART, months	1.01 (0.99 – 1.03)

HAART: highly active anti-retroviral therapy.

Therapy was scaled up to second line antiretroviral therapy due to treatment failure in four children.

### Adherence

The total number of children who were non-adherent to therapy was 35 (20%). The mean (SD) age of the children who were non-adherent was 150.7 (53.1) months, with 82.7% (29) of them being boys. A logistic regression analysis was done to determine the factors predicting non-adherence; after adjusting for age at diagnosis, gender, clinical stage of disease at diagnosis, whether parents are alive or not and duration of HAART; it was observed that the older the child more the chances of non-adherence (OR = 1.02, 95% CI: 1.004, 1.03, p = 0.008) and also there was a trend towards more chances of non-adherence in case the father of the child had expired (p = 0.06) as compared to children whose father was alive, irrespective of whether mother was alive or not.

### Adverse events

Adverse events were noted in 21 (12%) children. The adverse events observed during the follow up visits were pancreatitis due to stavudine (1 child), rash due to nevirapine (4 children), lipodystrophy due to stavudine (5 children), anemia due to zidovudine (6 children), hepatitis (4 children) and peripheral neuropathy (1 child). Antiretroviral treatment had to be changed in 20 children because of adverse events or tuberculosis. Treatment was changed from nevirapine based to efavirenz based regimen in 9 children (7 due to starting of antitubercular therapy, 2 due to NVP sensitivity), stavudine to zidovudine based regimen in 3 children (2 due to lipodystrophy, 1 due to pancreatitis), zidovudine to stavudine based therapy regimen in 8 children (6 due to anemia, 2 due to availability issues). The median time of changing of regime from initiation of HAART was 8 (IQR: 4, 22) months.

## Discussion

This cohort of 175 children gives us a good insight into the clinical and immunological characteristics of HIV-infected Indian children at the initiation of HAART and their response to treatment. The early diagnosis of children with HIV infection is imperative to initiate HAART before significant immunological compromise occurs [1],[16]. In our study, the children had a delayed presentation and more than half of HIV-infected children (57%) presented with severe immunological compromise. Resource limited settings seem to share this experience as seen in earlier Indian and African studies [3],[17],[18]. More stress on early diagnosis and encouragement of early health seeking behavior is necessary for a successful campaign against HIV-AIDS.

In this cohort of children, a large proportion (74.9%) were boys. In our setting the male: female disparity is commonly seen for most disease conditions suggesting a gender bias in health care seeking behaviors of the population [19].

After initiation of HAART, there was overall good immunological response with CD4 count more than doubled at 18 months post HAART. Similar immunological recovery both temporally and quantitatively has been reported by other studies [8]-[10],[18]. The sustained immunologic improvement in terms of CD4 count at least till 7 years after initiation of therapy, has also been reported from other long term follow-up study in children [20]. The viral load and ‘z’ scores for weight and height for age also improved with time in our study. Mulu et al. reported virological suppression in 87% children receiving HAART for a median of 24 months [21]. Patel et al. reported similar effects of protease inhibitor (PI) based HAART and NNRTI based HAART in children followed up for a median of 1 year [22].

There was an improvement in the overall clinical status of the children with fewer numbers of episodes of acute infections with time. This has been described in other studies in children [2],[4].

About five percent of our children were categorized as treatment failure based on clinical, immunological or virological criteria. Recent estimates of treatment failure and requirement of 2nd line HAART in low and middle income countries is around 3% [23]. The mean time to treatment failure in our study was found to be longer than the time to treatment failure reported in other studies [24]. Also, in contrast to the study by Bachaet al, treatment failure in our study was independent of the predictors like age at diagnosis, gender, initial CD4 count and duration of HAART [24]. The CD4 count trajectory was significantly associated with gender and age in our study. Female children showed better CD4 recovery compared to male children. Study conducted by Barry O et al., in Ghana reported similar finding of correlation between CD4 trajectory and gender [25].

In terms of safety, the antiretroviral therapy used in our children was safe with few adverse effects observed.

Self-reported adherence was about 80% in our patients as well as in other studies from India and Africa [26],[27]. Non-adherence in our children was associated with age of the child and death of father. Other studies probing into the correlates of adherence in an Indian setting have found orphanhood, longer duration of therapy to be amongst the factors predicting poor adherence [27].

Predictors of response to treatment include: age at initiation, immune status and viral load at time of initiation [22]. Ensuring excellent adherence to the HAART regimen is also crucial for ensuring long term effectiveness of HAART regimen. An adherence indicator based on timeliness of clinic attendance has been shown to strongly predict both virological response and drug resistance; the authors conclude that this indicator could help to timely identify non-adherent patients in settings where viral load monitoring is not available [28].

Globally, over the years, there has been tremendous progress in diagnosing and treating infants and children with HIV infection. However, in many low resource settings, the diagnosis and treatment of children with HIV infection still remains a challenge. The reasons for this vary from lack of technical expertise to inadequate availability of resources. Viral load is an important parameter for monitoring treatment response. There should be provision in the national guidelines to provide laboratory facilities for viral load testing to monitor the treatment response and also to detect the treatment failure.

The limitation of the study is that the causal association between CD4 count, viral load and other clinical parameters and overall treatment response cannot be accurately determined due to the retrospective nature of the study. Also, the viral load studies were not available for all the patients in our study. So, we could not accurately comment on the virological response and virological failure.

The strengths of our study lie in the large number of children followed up along with the long duration of follow-up of upto seven years.

## Conclusions

Our experience shows that NNRTI-based HAART in HIV-infected children is effective, safe and is associated with good immunological and virological response as well as improvement in growth parameters over a long term.

## Authors’ original submitted files for images

Below are the links to the authors’ original submitted files for images. Authors’ original file for figure 1 Authors’ original file for figure 2
