# Supplementary figures and images for: Outcome of highly active antiretroviral therapy in HIV- infected Indian children
Source: BMC Infect Dis. 2014 Dec 24;14:701. doi: 10.1186/s12879-014-0701-2 (PMC4297378; doi:10.1186/s12879-014-0701-2)

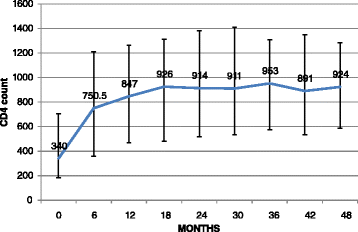

Supplement: Supplementary file 1 — Authors’ original file for figure 1 [file 12879_2014_701_MOESM1_ESM.gif]

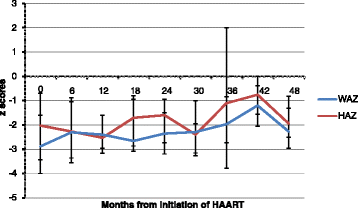

Supplement: Supplementary file 2 — Authors’ original file for figure 2 [file 12879_2014_701_MOESM2_ESM.gif]
